# Downregulation of *MicroRNA-193b-3p* Promotes Autophagy and Cell Survival by Targeting TSC1/mTOR Signaling in NSC-34 Cells

**DOI:** 10.3389/fnmol.2017.00160

**Published:** 2017-05-30

**Authors:** ChunYu Li, YongPing Chen, XuePing Chen, QianQian Wei, Bei Cao, HuiFang Shang

**Affiliations:** ^1^Department of Neurology, West China Hospital, Sichuan UniversityChengdu, China; ^2^West China Brain Research Center, West China Hospital, Sichuan UniversityChengdu, China

**Keywords:** *MicroRNA-193b-3p*, TSC1, mTORC1, cell death, autophagy

## Abstract

Amyotrophic lateral sclerosis (ALS) is a fatal neurodegenerative disease characterized by the death of upper and lower motor neurons. MicroRNAs (miRNAs) are reported to be closely related to the development of ALS. However, the precise functions of miRNAs in the pathogenesis of ALS remain largely unknown. In previous studies, we determined that *miRNA-193b-3p* was significantly downregulated in patients with sporadic ALS (sALS). Here, we observed that *miRNA-193b-3p* was downregulated in the SOD1^G93A^ mouse model of ALS and promoted cell death in NSC-34 cells. We further found that *miR-193b-3p* directly targeted tuberous sclerosis 1 (TSC1) to regulate mechanistic target of rapamycin complex 1 (mTORC1) activity. Downregulation of *miR-193b-3p* led to TSC1 increase accompanied with mTORC1 inactivation, and *vice versa*. Moreover, downregulation of *miR-193b-3p* promoted protective autophagy and cell survival in NSC-34 cells. In contrast, upregulation of *miR-193b-3p* activated mTORC1 signaling, leading to inhibition of autophagy and promotion of cell death. Taken together, our study suggests that downregulation of *miR-193b-3p* is required for cell survival by targeting TSC1/mTOR signaling in NSC-34 cells and provides a novel target for improving the clinical therapy of ALS.

## Introduction

Amyotrophic lateral sclerosis (ALS) is a common fatal motor neuron disease characterized by selective degeneration of upper and lower motor neurons (Boillee et al., 2006; Kiernan et al., 2011; Turner et al., 2011) leading to progressive muscle atrophy and weakness. ALS can be classified into sporadic ALS (sALS) or familial ALS (fALS). Several causative genes including chromosome 9 open reading frame 72 (*C9orf72*, DeJesus-Hernandez et al., 2011), Cu/Zn-superoxide dismutase *(SOD1*; Rosen et al., 1993), TAR DNA-binding protein *(TARDBP)* which encodes TDP43 (Sreedharan et al., 2008), and fused in sarcoma (FUS; Vance et al., 2009) have been shown to be involved in the development of ALS. Approximately 20% of fALS and 5% of sALS cases are caused by mutations in the *SOD1* gene (Majoor-Krakauer et al., 2003; Robberecht and Philips, 2013). Aberrant misfolded proteins resulting from *SOD1* mutation contribute to increased cellular stress and axon degeneration (Wilcox et al., 2009; Saccon et al., 2013). An animal model with overproduction of pathogenic human SOD1 protein develops late-onset progressive neurodegenerative disease (Saccon et al., 2013). However, the exact mechanisms and pathological processes responsible for the initiation and progression of motor neuron degeneration remain largely unknown.

Excessive cellular stress causes progressive damage to motor neurons (Turner and Atkin, 2006; Barber and Shaw, 2010). Accordingly, the neuronal defensive system must be evoked to protect neurons from death (Novoselov et al., 2013; Wang et al., 2015). Identification and investigation of ALS-relevant molecular alterations may aid in the identification of potential therapeutic targets. For example, SOD1 mutants specifically render vulnerable motor neurons dependent on endogenous neuroprotection signaling involving excitability and mechanistic target of rapamycin (mTOR; Leibinger et al., 2012; Saxena et al., 2013). mTOR signaling senses extracellular stimuli and regulates many biological processes, such as cell growth, energy metabolism and autophagy (Thomson et al., 2009; Laplante and Sabatini, 2012). mTOR is a PI3K-like serine/threonine protein kinase that is evolutionarily conserved in all eukaryotes (Dazert and Hall, 2011; Bordon, 2013). Dysregulation of mTOR signaling has been shown to be closely associated with cancers, metabolic diseases as well as neurodegenerative diseases. mTOR resides in two distinct complexes referred to as mTOR complex 1 (mTORC1) and mTOR complex 2 (mTORC2; Sarbassov et al., 2005). The tuberous sclerosis tumor suppressor complex (TSC), composed of TSC1 and TSC2, negatively regulates mTORC1 activity (Yang et al., 2016).

Notably, mTORC1 has been identified as a key regulator of autophagy inhibition (Kim et al., 2011). Autophagy could protect cells from death (Hara et al., 2006; Komatsu et al., 2006), especially in the case of motor neurons (Barmada et al., 2014). Autophagy is a highly conserved intracellular pathway involved in the elimination of proteins and organelles by lysosomes. Autophagy is now recognized as an arbiter of neuronal survival and death decisions in neurodegenerative diseases (Banerjee et al., 2010). In ALS, defective autophagy has also been implicated in the accumulation of ubiquitinated TDP-43 inclusions and motor neuron degeneration (Caccamo et al., 2009). Moreover, some studies report that autophagic clearance of mutant SOD1 exert protective effect against motor neuron loss in an ALS mouse model (Crippa et al., 2010). Therefore, autophagy seems to be protective for the survival of motor neurons in ALS. However, the precise role and regulatory factors of autophagy in motor neuron degeneration in ALS remain to be determined.

MicroRNAs (miRNAs) are small, single-stranded, noncoding RNAs that consist of approximately 18–22 nucleotides and can regulate protein expression either by translational inhibition or targeted mRNA cleavage (Bartel, 2009; Guo et al., 2010). Growing evidence suggests that miRNAs play an important role in neurodegenerative diseases, including ALS (Akerblom et al., 2012; Goodall et al., 2013; Zhu et al., 2013; Parisi et al., 2016). Furthermore, mutations of *TARDBP* and *FUS* in ALS, both of which are closely related to miRNA processing, give rise to more links between ALS and miRNAs (Morlando et al., 2012; Di Carlo et al., 2013). A single miRNA may have multiple mRNA targets, allowing it to be involved in diverse pathological processes (Filipowicz et al., 2008). However, the exact mechanisms and pathological processes responsible for the initiation and progression of motor neuron degeneration by miRNAs remain largely unknown.

In previous studies, we investigated the miRNA expression profiles of Chinese sALS patients to explore new potential biomarkers for the diagnosis of ALS. We noted that *miR-193b-3p* was downregulated in sALS patients and provided high diagnostic accuracy for sALS (Chen et al., 2016). Here, we used NSC-34 cells to investigate the fundamental functions of *miR-193b-3p* in the development of ALS. We found that *miR-193b-3p* was downregulated in mouse model of ALS and promoted cell death in NSC-34 cells. Our work suggests that downregulation of *miR-193b-3p* is required for cell survival by targeting TSC1/mTOR signaling to promote autophagy. These findings may inform novel therapeutic targets for ALS.

## Materials and Methods

### Reagents and Chemicals

For cell culture *in vitro*, Dulbecco’s Modified Eagle’s Medium (DMEM), trypsin and Fetal Bovine Serum (FBS) were obtained from GIBCO Invitrogen (Carlsbad, CA, USA). The anti-TSC1, TSC2, p70S6K, pp70S6K (Thr389), 4EBP1, p-4EBP1 (Thr37/46) and GAPDH antibodies were purchased from Cell Signaling Technology (Beverly, MA, USA). p62 and LC3 antibodies were purchased from Novus Biological Inc. (USA). The mTOR inhibitor Torin1 was purchased from Tocris Bioscience. All other reagents were obtained from Sigma-Aldrich with the highest purity available.

### Cell Culture and Treatment

For *in vitro* experiments, we used the NSC-34 cell line provided by Dr. N.R. Cashman (University of Toronto, Toronto, Canada; Cashman et al., 1992). NSC-34 cells were cultured in DMEM (Gibco) containing 10% (FBS, Gibco), 100 U/mL penicillin, 100 mg/mL streptomycin (Invitrogen) at 37°C in a 95% air/5% CO_2_ atmosphere at constant humidity. Transfection of *miR-193b-3p* mimics, inhibitors and scrambled sequences (Ribobio, Guangzhou, China) were carried out when the cell confluent was 80%–90% using RNAiMAX (Invitrogen) according to the manufacturer’s instructions.

### SOD1^G93A^ Transgenic Mice

SOD1^G93A^ transgenic mice were purchased from the Jackson Lab (Bar Harbor, ME, USA). Animal care and procedures were performed in accordance with the Laboratory Animal Care Guidelines approved by the Animal Care and Use Committee of Sichuan University West-China Hospital.

### Luciferase Reporter Assay

The TSC1 3′UTR was cloned into the *Xba*I and *EcoR*I sites of the pMIR-REPORT luciferase vector (Ambion, USA), and the reconstituted plasmid was named pWT. The TSC1 3′UTR mutations were introduced using the Multisite-Quickchange kit (Stratagene, CA, USA) according to the manufacturer’s protocol and cloned into the pMIR-REPORT luciferase vector (Ambion, USA), and the reconstituted plasmid was named pMUT. All inserted or mutated sequences were confirmed by sequencing. NSC-34 cells were transfected with *miR-193b-3p* mimics and pWT using Lipofectamine RNAiMAX transfection reagent according to the manufacturer’s instructions. *miR-193b-3p* mimics and pMT, or miRNA negative control (miR-NC) and pWT, or miR-NC and pMT were also transfected into NSC-34 cells as controls. Luciferase activity was measured in cell lysates 48 h after transfection using the Dual-Light^®^ Luminescent Reporter Gene Assay kit (Applied Biosystems, CA, USA).

### miRNA Extraction and RT-qPCR Assay

Total miRNA was extracted and collected from cells using the miRNeasy Mini Kit (Qiagen, Germany) according to the manufacturer’s protocol. Isolated miRNAs were reverse-transcribed to complementary DNA (cDNA) using a miScript II reverse transcription kit (Qiagen, Germany) with the standard protocol. Quantitative real-time PCR was carried out with the miScript miRNA PCR Array (Qiagen, Germany) using the SYBR-green-based real-time PCR (RT-PCR) method on a Bio-Rad PCR machine according to the manufacturer’s protocol. The primers for miRNAs were purchased from Ribobio, China. U6 rRNA was used as an internal control. Data analysis was performed using the 2^−ΔΔCt^ method.

### RNA Extraction and RT-qPCR Assay

Total RNAs were extracted from cultured cells using Trizol reagent (Invitrogen, USA). cDNA was synthesized from 2 μg of total RNA according to the manufacturer’s instructions (Thermo Fisher Scientific, USA). Quantitative real-time PCR was performed using the Bio-Rad iQ5 system (Bio-Rad, USA), and the relative gene expression was normalized to the internal control GAPDH. Primer sequences for SYBR-green probes of target genes were as follows, and data analysis was performed using the 2^−ΔΔCt^ method.

TSC1: 5′-ATGGCCCAGTTAGCCAACAT-3′ and 5′-CAGAATTGAGGGACTCCTT GAAG-3′;
GAPDH: 5′-AGGTCGGTGTGAACGGATTTG-3′ and 5′-TGTAGACCATGTAGTT GAGGTCA-3′;

### Protein Extractions and Western Blots

To extract total proteins, cultured NSC-34 cells were sonicated with lysis buffer (2% SDS with protease and phosphatase inhibitors). The protein concentration of each extract was measured by the BCA Protein Assay kit (Thermo Scientific Pierce). Equal amounts of denatured proteins (~20 μg) from each extract were separated by SDS-PAGE. Proteins were transferred onto PVDF membranes following standard procedures. The membranes were blocked with 5% skimmed milk in TBST (TBS with 0.1% Tween 20, pH 7.6) for 1 h at room temperature on a rocker and then incubated with various antibodies diluted in TBST (1:1000) at 4°C, overnight. The membranes were then washed three times with TBST for 10 min each wash, and the membranes were incubated with appropriate secondary antibodies diluted in TBST (1:10,000 for both the goat anti-rabbit and goat anti-mouse IgG antibodies) for 2 h at room temperature. The membranes were washed three times with TBST at room temperature for 10 min. Proteins were then detected with ECL reagent (Thermo Scientific/Pierce, Rockford, IL, USA), and the membranes were exposed to film (Kodak). Films were scanned, and optical densities were quantified using ImageJ software.

### Cell Viability Assay

A Cell Counting Kit-8 (CCK-8; Dojindo, Japan) assay was used to determine NSC-34 cell viability. Cells were seeded in a 96-well plate at a density of approximately 2–4 × 10^3^ cells *per* well in 200 μl of culture medium and treated as designated. The absorbance was measured in a microplate reader (Gene Company Limited, China) at a wavelength of 450 nm.

### Annexin V-FITC/PI Apoptosis Assay

For apoptosis examinations, we used the Annexin V-FITC/PI apoptosis assay. NSC-34 cells were digested into single cell suspensions using EDTA-free trypsin, and cells were stained according to the instructions provided with the Annexin V-FITC/PI Apoptosis Detection kit (KeyGen, Nanjing, China). The cells were analyzed after 20 min by flow cytometry.

### GFP-LC3 Puncta Imaging

To assay autophagic status, NSC-34 cells were transfected with GFP-LC3 plasmids and *miR-193b-3p* mimics, inhibitors or scrambled sequence. Cells were grown on glass coverslips and treated as designated. Cells were fixed with 4% paraformaldehyde and 4% sucrose and permeabilized with 0.1% Triton X-100 for 10 min. Finally, cells were rinsed and mounted on cover glasses with Prolong Gold anti-fade reagent with 4′,6-diamidino-2-phenylindole (DAPI; Invitrogen, USA) and visualized using an Olympus IX 81 (Olympus, Tokyo, Japan) microscope.

### Statistical Analysis

All quantitative results of western blots, real-time PCR and cell assays were presented as the mean and standard error of the mean (SEM) from at least three independent experiments and analyzed by SPSS 22 Package (SPSS, USA). P values were calculated using two-tailed, unpaired Student’s *t* test or analysis of variance (ANOVA) with an least significant differences (LSD) post-test analysis, and the values 0.05 (*), 0.01 (**) and 0.001 (***) were assumed as the level of significance for the statistic tests carried out.

## Results

### *miR-193b-3p* Is Downregulated in the Mouse Model of ALS and Promotes Cell Death in NSC-34 Cells

**Figure 1:**
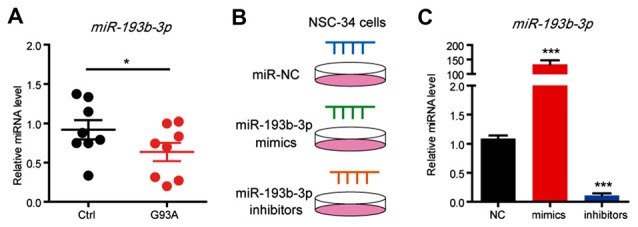
*miR-193b-3p* is downregulated in the mouse model of amyotrophic lateral sclerosis (ALS).** (A)** qRT-PCR results show the miRNA levels of *miR-193b-3p* in the spinal cord of SOD1^G93A^ mutants compared with controls (~90 days). The results were averages of eight pairs of littermate mice. Data represent the mean ± standard error of the mean (SEM). *P < 0.05 vs. controls. **(B,C)** qRT-PCR results show the miRNA levels of *miR-193b-3p* in NSC-34 cells transfected with *miR-193b-3p* mimics, inhibitors or scrambled sequence. The results were averages of four independent experiments. Data represent the mean ± SEM. ***P < 0.001 vs. controls.

**Figure 2:**
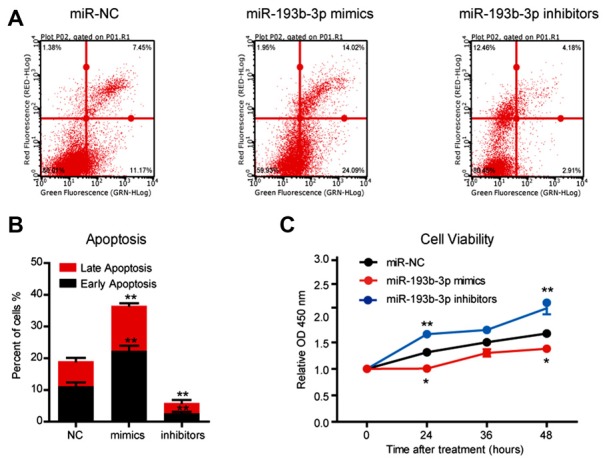
*miR-193b-3p* promotes cell death in NSC-34 cells. **(A,B)** The results of flow cytometry assays and quantification of cell apoptosis using FITC/PI staining in NSC-34 cells transfected with *miR-193b-3p* mimics, inhibitors or scrambled sequence. The results were averages of three independent experiments. Data represent the mean ± SEM. **P < 0.01 vs. controls. **(C)** The results of Cell Counting Kit-8 (CCK-8) assay indicate cell viability in NSC-34 cells transfected with *miR-193b-3p* mimics, inhibitors or scrambled sequence. The results were averages of three independent experiments. Data represent the mean ± SEM. *P < 0.5 and **P < 0.01 vs. control.

In a previous study, we demonstrated that *miR-193b-3p* was downregulated and might be a candidate miRNA in the pathogenesis of ALS (Chen et al., 2016). To confirm the pathogenic role of downregulation of *miR-193b-3p* in ALS, we further examined the expression of *miR-193b-3p* in the SOD1^G93A^ ALS mouse model (Ferrante et al., 1997; Chiu et al., 2008). The results showed that the expression of *miR-193b-3p* was also downregulated in the spinal cord of SOD1^G93A^ ALS mice compared with wild-type controls (~90 days; Figure 1A). We also noted that *miR-193b-3p* was decreased in the spinal cord of ALS mice from ~90 days to ~150 days (Supplementary Figure S1A). To investigate the role of *miR-193b-3p*, we transfected NSC-34 hybrid mouse motor neuron-like cells with mimics or inhibitors of *miR-193b-3p* (Figure 1B). qRT-PCR results showed that the expression of *miR-193b-3p* was dramatically induced by *miR-193b-3p* mimics and significantly blocked by its inhibitors (Figure 1C). To investigate the role of *miR-193b-3p* in cell survival, we detected cell apoptosis in NSC-34 cells by flow cytometry assay. The results showed that *miR-193b-3p* overexpression induced either early or late apoptosis in NSC-34 cells, while its inhibition exhibited opposite effects (Figures 2A,B). Furthermore, we confirmed this finding by investigating cell viability using the CCK-8 assay. The results showed that cell viability was decreased by *miR-193b-3p* overexpression and increased by its inhibition (Figure 2C). Therefore, we propose that upregulation of *miR-193b-3p* could promote cell death in NSC-34 cells.

### *miR-193b-3p* Directly Targets TSC1 and Regulates mTORC1 Activity in NSC-34 Cells

**Figure 3:**
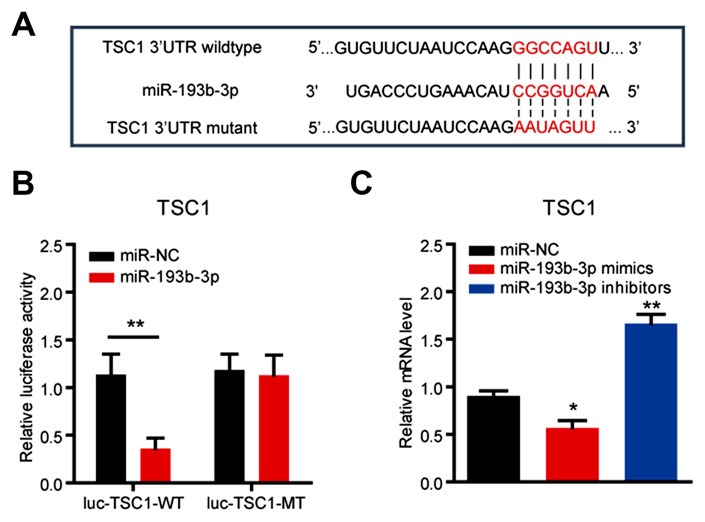
*miR-193b-3p* directly targets TSC1. **(A)** Sequence analysis of *miR-193b-3p* mature miRNA binding with the 3′-UTR of TSC1. **(B)** Luciferase reporter assay shows a reduction of luciferase activity in NSC-34 cells with wild-type TSC1 3′UTR (luc-TSC1-WT) plasmids. The results were averages of three independent experiments. Data represent the mean ± SEM. **P < 0.01 vs. controls. **(C)** qRT-PCR results indicate the mRNA levels of TSC1 in NSC-34 cells transfected with *miR-193b-3p* mimics, inhibitors or scrambled sequence (100 nM, respectively). The results were averages of four independent experiments. Data represent the mean ± SEM. *P < 0.5 and **P < 0.01 vs. controls. miR-NC, miRNA negative control.

**Figure 4:**
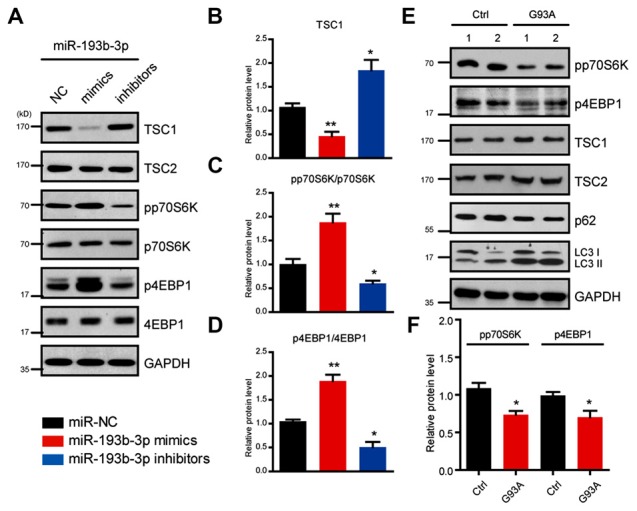
*miR-193b-3p* activates mTOR complex 1 (mTORC1) signaling by decreasing TSC1 in NSC-34 cells.** (A–D)** Western blots and quantification to show the protein levels of TSC1/2 and mTORC1 indicators (pp70S6K and p4EBP1) in NSC-34 cells transfected with *miR-193b-3p* mimics, inhibitors or scrambled sequence. The results were averages of four independent experiments. Data represent the mean ± SEM. *P < 0.5 and **P < 0.01 vs. controls. **(E,F)** Western blots and quantification to show the protein levels of mTORC1 indicators (pp70S6K and p4EBP1), TSC1/2, p62 and LC3 in the spinal cord of SOD1^G93A^ mutants compared with controls. The results were averages of three pairs of littermate mice. Data represent the mean ± SEM. *P < 0.05 vs. controls.

To identify the potential targets of *miR-193b-3p* in humans, we first screened the targets of *miR-193b-3p* by sequence analysis. The results showed that TSC1, a well-known regulator of mTORC1 signaling was a potential candidate of *miR-193b-3p* in NSC-34 cells (Figure 3A). By luciferase reporter assay, we confirmed the targeting sites of *miR-193b-3p* within TSC1 (Figure 3B). qRT-PCR results showed that the relative expression of *TSC1* was dramatically decreased by *miR-193b-3p* overexpression (mimics) and increased by its inhibition (inhibitors; Figure 3C). We also analyzed other potential targets of *miR-193b-3p* including Pten, Pfn1 and Prkca (Supplementary Figure S1B,C). To focus on how *miR-193b-3p* regulated TSC1, we further investigated TSC1 protein expression levels and mTORC1 activity in NSC-34 cells. Western blot results showed that the protein level of TSC1 was dramatically reduced by *miR-193b-3p* mimics and induced by its inhibitors (Figures 4A,B). Consistently, the indicators of mTORC1 signaling, pp70S6K and p4EBP1, were both increased by *miR-193b-3p* mimics and decreased by its inhibitors (Figures 4A,C,D). To confirm that *miR-193b-3p* mimics increased p70S6K and 4EBP1 phosphorylation in mTOR dependent manner, we applied Torin1 (a specific ATP-competitive inhibitor of mTOR) to NSC-34 cells transfected with *miR-193b-3p* mimics and examined the phosphorylation of p70S6K and 4EBP1. The results showed that Torin1 could block the *miR-193b-3p* mimic-induced phosphorylation of p70S6K and 4EBP1, suggesting that *miR-193b-3p* mimics increase p70S6K and 4EBP1 phosphorylation in an mTOR-dependent manner (Supplementary Figure S2A). To further prove that *miR-193b-3p* targets TSC1 to regulate mTOR signaling, we applied *miR-193b-3p* inhibitors to TSC1 knockdown NSC-34 cells. The results showed that *miR-193b-3p* inhibitors could neither increase TSC1 protein nor decrease mTORC1 activity in TSC1 knockdown cells (Supplementary Figure S2B). Therefore, we propose that *miR-193b-3p* indeed targets TSC1 to control mTOR signaling.

Next, we examined the activity of mTORC1 signaling in SOD1^G93A^ mutant mice and found that pp70S6K and p4EBP1, two important indicators of mTORC1 signaling, were both decreased in the spinal cord of SOD1^G93A^ mutant mice compared with controls, consistent with the inhibition manipulation of *miR-193b-3p* (Figures 4E,F). We also noted that autophagy was activated in the spinal cord of SOD1^G93A^ mutant mice, as indicated by the increase of autophagy markers LC3 and p62 (Figure 4E). Taken together, our data revealed that *miR-193b-3p* was a positive regulator of mTORC1 signaling and directly targeted TSC1 to control mTORC1 activity in NSC-34 cells.

### *miR-193b-3p* Inhibits Autophagy in NSC-34 Cells

**Figure 5:**
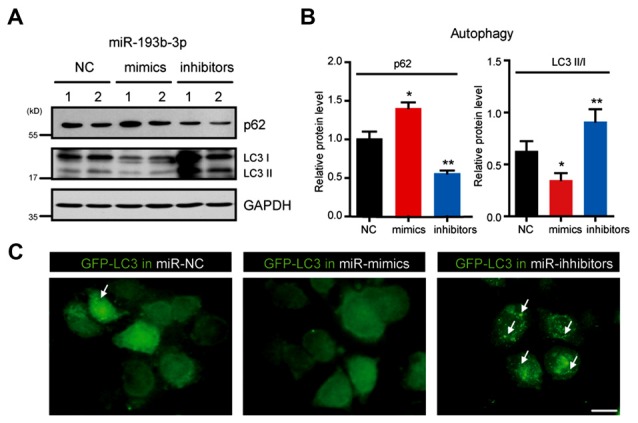
*miR-193b-3p* inhibits autophagy in NSC-34 cells. **(A,B)** Western blots and quantification to show the protein levels of p62 and LC3 (II/I) in NSC-34 cells transfected with *miR-193b-3p* mimics, inhibitors or scrambled sequence. The results were averages of four independent experiments. Data represent the mean ± SEM. *P < 0.5 and **P < 0.01 vs. controls. **(C)** Representative images of GFP-LC3 puncta (white arrows) in NSC-34 cells transfected with *miR-193b-3p* mimics, inhibitors or scrambled sequence. Scale bar, 10 μm.

The current understanding of TSC1/mTOR in cell survival involves multiple aspects, including cell proliferation, metabolism and autophagy (Matsuzawa et al., 2015). mTOR is widely accepted as a negative regulator of autophagy (Kim et al., 2011). Autophagy is now recognized as protective for neuronal survival in neurodegenerative diseases (Hara et al., 2006; Komatsu et al., 2006; Lee, 2012). To investigate whether *miR-193b-3p* contributes to cell death through impairing autophagy, we examined the autophagic status under the conditions of *miR-193b-3p* overexpression (mimics) and inhibition (inhibitors) in NSC-34 cells. The results showed that *miR-193b-3p* inhibition enhanced autophagy (indicated by decreased p62 and increased ratio of LC3II/I) in NSC-34 cells. We also found that *miR-193b-3p* mimics increased p62 protein expression and decreased the ratio of LC3II/I in NSC-34 cells (Figures 5A,B, and Supplementary Figure S2C). To confirm the effect of *miR-193b-3p* on autophagy, we transfected GFP-LC3 into NSC-34 cells and examined whether *miR-193b-3p* mimics or inhibitors could alter GFP-puncta formation. Images showed that *miR-193b-3p* inhibitors dramatically increased the formation of GFP-LC3 puncta, whereas *miR-193b-3p* mimics decreased the formation of GFP-LC3 puncta (Figure 5C). Taken together, these results indicate that *miR-193b-3p* negatively regulates autophagy in NSC-34 cells.

## Discussion

**Figure 6:**
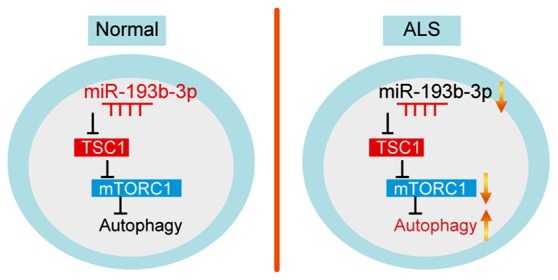
Model. Schematic representation highlighting the role of *miR-193b-3p* in the development of ALS. *miR-193b-3p* directly targets TSC1 to regulate mTORC1 activity. Inhibition of *miR-193b-3p* leads to TSC1 increase and mTORC1 inactivation, resulting in enhanced autophagy.

Increasing evidence suggests that miRNAs play an important role in the development of ALS. Ours and other previous studies have assessed miRNA expression profiles in ALS patients. Despite these findings, it is still unknown how alterations of specific miRNAs contribute to the development of ALS. In this study, we tried to clarify whether *miR-193b-3p*, a downregulated miRNA in ALS patients reported in our previous study (Chen et al., 2016), involves in cell survival by targeting TSC1/mTOR signaling in autophagy (Figure 6). Our findings suggest that downregulation of *miR-193b-3p* is required for cell survival and provide novel molecular mechanisms for the detection and treatment of ALS.

Interestingly, *miRNA-193a-3p* and *miRNA-193a-5p* could directly target and downregulate the ERBB4/PIK3R3/mTOR/ S6K2 signaling pathway (Yu et al., 2015). It has been reported that ERBB4 and S6K2 are the direct targets of *miR-193a-3p* and that PIK3R3 and mTOR are the direct targets of *miR-193a-5p* in non-small-cell lung cancer (Yu et al., 2015). In this study, we obtained evidence that *miR-193b-3p* targets the upstream factor of mTOR (TSC1) to counteract the effect of *miR-193a*. Therefore, we propose that these effects may be beneficial for the precise regulation of mTOR signaling, and *miR-193b-3p* directly targets TSC1 and modulates mTOR activity. To clarify the role of *miR-193b-3p*/TSC1/mTOR axis in the development of ALS, we utilized NSC-34 cells as an *in vitro* model. NSC-34 is a hybrid motor neuron-like cell line produced by the fusion of neuroblastoma with mouse motor neuron-enriched primary spinal cord cells (Eggett et al., 2000; Matusica et al., 2008; Maier et al., 2013), and it is widely used in studies of ALS *in vitro*. We found that *miR-193b-3p* regulates autophagy for cell survival in NSC-34 cells through TSC1/mTOR. These results at least reveal potential roles of *miR-193b-3p* in cell death of motor neurons in the development of ALS.

Neurodegenerative diseases such as Parkinson’s disease, Alzheimer’s disease, Huntington’s disease, and ALS are associated with the permanent loss of neuronal structure and function (Laplante and Sabatini, 2012). Recently, the protective role of mTOR has been noted in neuronal degeneration. Genetic and pharmacological evidence has shown that deletion of TSC1, a negative regulator of mTOR signaling, led to constitutive activation of mTOR, neuroprotective effects and potently enhanced axon regeneration (Park et al., 2008). Moreover, mTOR activity in motor neurons influenced the progression rates of motor dysfunction, muscle denervation and cell death, suggesting that mTOR signaling is required for endogenous neuroprotection to counteract disease progression in fALS (Saxena et al., 2013). However, mTORC1 signaling coordinately activates anabolic processes, such as protein synthesis, while inhibiting the cellular catabolism of autophagy (Chan, 2009). Autophagy could protect cells from death (Hara et al., 2006; Komatsu et al., 2006), especially in motor neurons (Barmada et al., 2014). Accumulating evidence indicates that maintaining a balanced autophagic flux is essential in neuronal physiology. Neurons are highly specialized cells that depend on dynamic cellular processes for their proper function. Once neurons encounter stress, they develop multiple cellular processes to resist these pressures, such as autophagy (Nikoletopoulou et al., 2015). Autophagy is a tightly regulated cellular degradation pathway, which is often defective or hyperactive in neurodegenerative diseases (Laplante and Sabatini, 2012). Currently, accumulating evidence suggests that autophagy is deregulated in neurodegenerative diseases and may play key roles in the etiology of these pathologies (Rubinsztein, 2006). Many studies in cellular and animal models of ALS indicate enhanced autophagy activity in ALS (Morimoto et al., 2007; Sasaki, 2011), in addition to the occurrence of autophagy-mediated clearance of mutant SOD1 and TDP-43 (Nassif et al., 2010). For example, the number of autophagic vacuoles is significantly increased in the motor neurons of the spinal cords of SOD1^G93A^ mice compared with controls (Massey et al., 2006). Therefore, it is predicted that mechanistic target of rapamycin (mTORC1) activity should be downregulated in ALS to meet the demand of increased autophagy. Our findings confirmed that *miR-193b-3p* is downregulated in ALS patients and mouse models, accompanied by decreased mTORC1 activity. The co-reduction of *miR-193b-3p* and mTORC1 activity promotes autophagy to protect cells from death.

Autophagy plays an important role in neurodegenerative diseases. However, the contribution of autophagy to the pathology of ALS has not yet been fully elucidated. Although autophagic alteration has been confirmed in ALS patients and experimental models, it remains controversial whether activating autophagy is beneficial or detrimental for motor neuron degeneration. Studies suggest that defects in autophagic flux or specific autophagy-regulatory processes, rather than simple induction of autophagy, may contribute to motor neuron degeneration (Banerjee et al., 2010). In this study, we found that *miR-193b-3p* targets TSC1 and thus modulates mTOR activity, which negatively regulates autophagy. Inhibition of *miR-193b-3p* downregulates mTOR activity and activates autophagy, which may protect cells from death. Therefore, we propose that the effect of *miR-193b-3p* on TSC1/mTOR signaling is fundamentally important to cell survival in ALS development. However, further longitudinal study in ALS patients will be helpful to confirm these results. Nevertheless, manipulating the autophagy process is a complicated dilemma. It is anticipated that more specific autophagic regulators will be discovered and deeper understanding of autophagy biology will be obtained in the near future, which will help decode the mystery of autophagy in ALS pathogenesis and the therapeutic value of autophagy modulators for this devastating disease.

In conclusion, our results suggest that *miR-193b-3p* directly targets TSC1 to regulate mTORC1 activity. Inhibition of *miR-193b-3p* leads to increased TSC1 and mTORC1 inactivation. Increased *miR-193b-3p* activates mTORC1 signaling, inhibits autophagy and thus promotes cell death. Moreover, the downregulation of *miR-193b-3p* could be a potential biomarker for the detection of ALS development. Taken together, our work supports the hypothesis that *miR-193b-3p* decrease is required for cell survival by improving autophagy through the TSC1/mTOR pathway and might inform the development of early therapeutic strategies in ALS.

## Author Contributions

HS conceived and designed the research. CL collected, analyzed and interpreted the data and drafted the manuscript. CL, YC, QW and BC performed the experiments. HS, YC and XC revised the article critically. All authors approved the manuscript.

## Conflict of Interest Statement

The authors declare that the research was conducted in the absence of any commercial or financial relationships that could be construed as a potential conflict of interest.

## Supplementary Material

FIGURE S1 *miR-193b-3p* is downregulated in the mouse model of ALS, related to Figures 1, 3. **(A)** qRT-PCR results to show the miRNA levels of *miR-193b-3p* in the spinal cord of SOD1^G93A^ mutants compared with controls, from 60 days to 150 days. The results were averages of eight pairs of littermate mice. Data represent the mean ± SEM. **P* < 0.05 vs. controls. **(B)** Sequence analysis of *miR-193b-3p* mature miRNA binding with 3′-UTR of Pten, Pfn1 and Prkca. **(C)** Luciferase reporter assay to show the reduction of luciferase activity in NSC-34 cells with 3′-UTR of Pten, Pfn1 and Prkca plasmids. The results were averages of three independent experiments. Data represent the mean ± SEM. ****P* < 0.001 vs. controls.

FIGURE S2 *miR-193b-3p* regulates TSC1/mTOR signaling and autophagy in NSC-34 cells, related to Figures 4, 5. **(A)** Western blots show the protein levels of mTORC1 indicators (pp70S6K and p4EBP1) in NSC-34 cells by *miR-193b-3p* mimics with treatment of Torin1 (200 nM for 12 h). **(B)** Western blots show the protein levels of TSC1 and mTORC1 indicators (pp70S6K), and autophagy markers (p62 and LC3) in NSC-34 cells by *miR-193b-3p* inhibitors with TSC1 knockdown. **(C)** Original blots of Figure 5A.

## Abbreviations

ALS: amyotrophic lateral sclerosis
*C9orf72*: chromosome 9 open reading frame 72
fALS: familial amyotrophic lateral sclerosis
*FUS*: fused in sarcoma
miRNA: microRNA
miR-NC: miRNA negative control
mTOR: mechanistic target of rapamycin
mTORC1: mTOR complex 1
mTORC2: mTOR complex 2
sALS: sporadic amyotrophic lateral sclerosis
*SOD1*: superoxide dismutase 1
*TARDBP*: TAR DNA-binding protein
TSC: tuberous sclerosis complex.
