# Supplementary figures and images for: Downregulation of MicroRNA-193b-3p Promotes Autophagy and Cell Survival by Targeting TSC1/mTOR Signaling in NSC-34 Cells
Source: Front Mol Neurosci. 2017 May 30;10:160. doi: 10.3389/fnmol.2017.00160 (PMC5447700; doi:10.3389/fnmol.2017.00160)

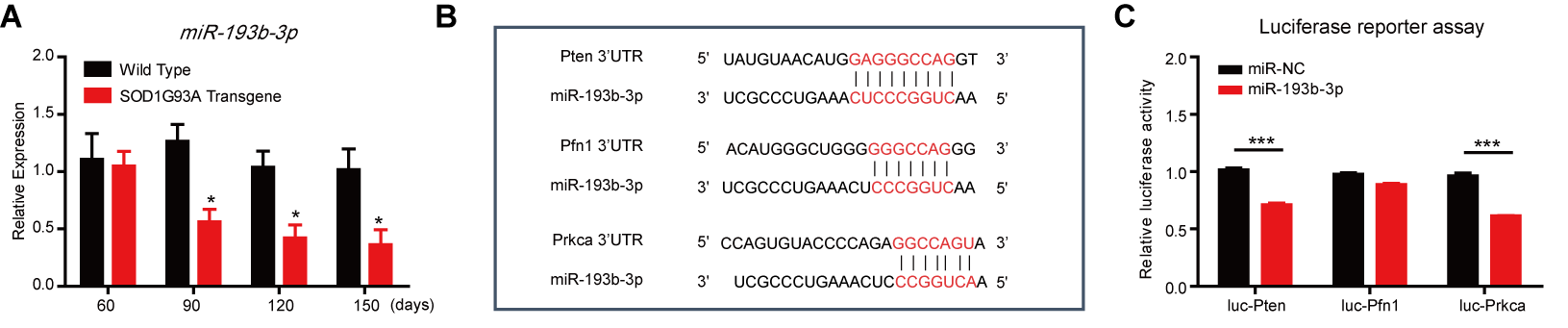

Supplement: FIGURE S1 — miR-193b-3p is downregulated in the mouse model of ALS, related to Figures 1, 3. (A) qRT-PCR results to show the miRNA levels of miR-193b-3p in the spinal cord of SOD1G93A mutants compared with controls, from 60 days to 150 days. The results were averages of eight pairs of littermate mice. Data represent the mean ± SEM. *P < 0.05 vs. controls. (B) Sequence analysis of miR-193b-3p mature miRNA binding with 3′-UTR of Pten, Pfn1 and Prkca. (C) Luciferase reporter assay to show the reduction of luciferase activity in NSC-34 cells with 3′-UTR of Pten, Pfn1 and Prkca plasmids. The results were averages of three independent experiments. Data represent the mean ± SEM. ***P < 0.001 vs. controls. [file Image_1.tif]

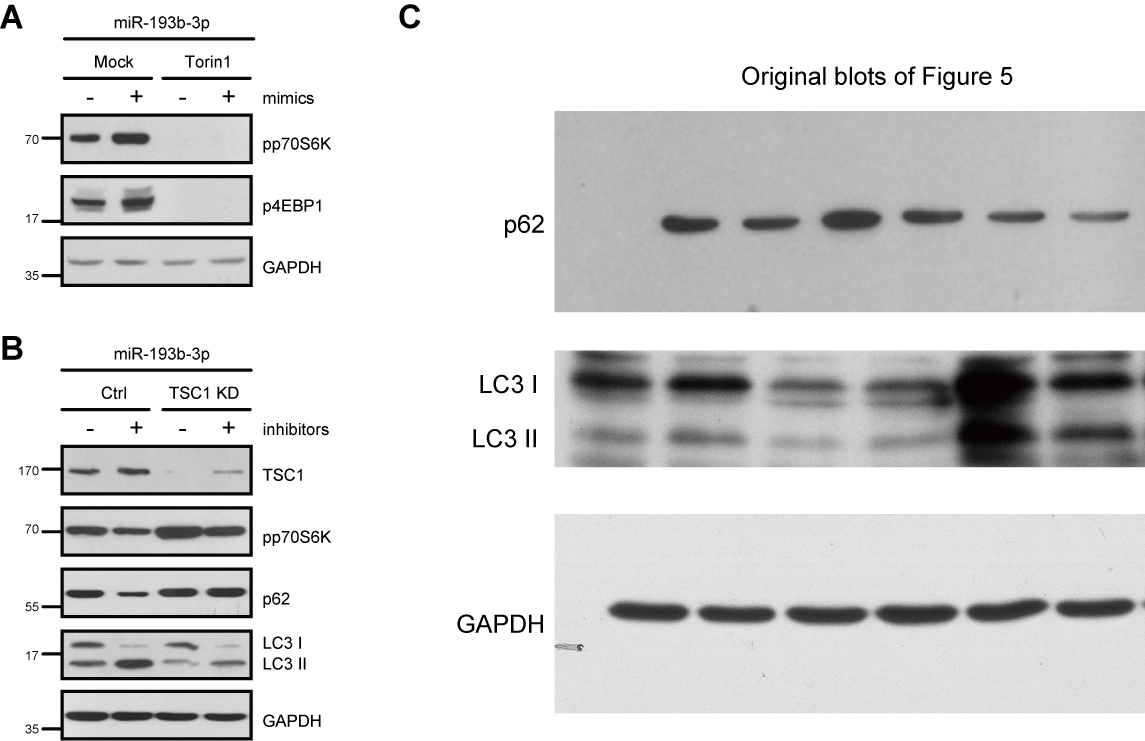

Supplement: FIGURE S2 — miR-193b-3p regulates TSC1/mTOR signaling and autophagy in NSC-34 cells, related to Figures 4, 5. (A) Western blots show the protein levels of mTORC1 indicators (pp70S6K and p4EBP1) in NSC-34 cells by miR-193b-3p mimics with treatment of Torin1 (200 nM for 12 h). (B) Western blots show the protein levels of TSC1 and mTORC1 indicators (pp70S6K), and autophagy markers (p62 and LC3) in NSC-34 cells by miR-193b-3p inhibitors with TSC1 knockdown. (C) Original blots of Figure 5A. [file Image_2.tif]
